# Enhancing the Screening Efficiency of Breast Cancer by Combining Conventional Medical Imaging Examinations With Circulating Tumor Cells

**DOI:** 10.3389/fonc.2021.643003

**Published:** 2021-05-19

**Authors:** Yang Gao, Wan-Hung Fan, Chaohui Duan, Wenhe Zhao, Jun Zhang, Xixiong Kang

**Affiliations:** ^1^Key Laboratory for Biomechanics and Mechanobiology of Ministry of Education, School of Biological Science and Medical Engineering, Beihang University, Beijing, China; ^2^Beijing Advanced Innovation Center for Biomedical Engineering, Beihang University, Beijing, China; ^3^Department of Clinical Medical Affairs, Hangzhou Watson Biotech, Hangzhou, China; ^4^Department of Clinical Laboratory, Sun Yat-Sen Memorial Hospital, Sun Yat-Sen University, Guangzhou, China; ^5^Department of Oncology, Sir Run Run Shaw Hospital, Zhejiang University School of Medicine, Hangzhou, China; ^6^Department of Clinical Laboratory, Sir Run Run Shaw Hospital, Zhejiang University School of Medicine, Hangzhou, China

**Keywords:** breast cancer, circulating tumor cells, mammogram, ultrasound, screening

## Abstract

**Purpose:**

Ultrasound (US) and mammogram (MMG) are the two most common breast cancer (BC) screening tools. This study aimed to assess how the combination of circulating tumor cells (CTC) with US and MMG would improve the diagnostic performance.

**Methods:**

CTC detection and imaging examinations, US and MMG, were performed in 238 treatment-naive BC patients, 217 patients with benign breast diseases (BBD), and 20 healthy females. Correlations of CTC, US and MMG with patients’ clinicopathological characteristics were evaluated. Diagnostic performances of CTC, US and MMG were estimated by the receiver operating characteristic curves.

**Results:**

CTC, US and MMG could all distinguish BC patients from the control (p < 0.0001). Area under curve (AUC) of CTC, US and MMG are 0.855, 0.861 and 0.759, respectively. While US has the highest sensitivity of 0.79, CTC and MMG have the same specificity of 0.92. Notably, CTC has the highest accuracy of 0.83. Combination with CTC increases the AUC of US and MMG to 0.922 and 0.899, respectively. Combining MMG with CTC or US increases the sensitivity of MMG to 0.87, however “CTC + MMG” has a higher specificity of 0.85. “CTC + US” performs the best in BC diagnosis, followed by “CTC + MMG” and then “US + MMG”.

**Conclusion:**

CTC can be used as a diagnostic aid for BC screening. Combination with CTC increases the diagnostic potency of conventional BC screening imaging examinations, US and MMG, in BC diagnosis, especially for MMG.

## Introduction

Breast cancer (BC) is the most frequently diagnosed cancer and the leading cause of cancer-related death in women worldwide (1). Every year there are more than 2 million of newly diagnosed cases and more than 630,000 people died of BC globally (1). About 12.4% of women (1 in 8) will develop BC at some point in their lives (1, 2). Although the incidence and mortality rates of BC are ranked 120 and 163 respectively in the world, BC is still the most common cancer among females in China (2, 3). It is estimated that 304,000 BC cases were newly diagnosed and approximately 69,900 women died of BC in China in 2015 (3). BC mortality rates have declined over the passing decades in developed countries such as the United States and United Kingdom, but death from BC in China is still slowly increasing (2). BC is generally diagnosed through either screening or a symptom (breast swelling or a palpable mass) that leads to a medical examination (4). The decline in BC mortality is mainly attributed to a combination of advances in prevention or screening and improved treatment methods (4). The aims of cancer prevention and screening are to reduce cancer incidence by removing carcinogenic factors from the daily-life and to identify asymptomatic patients at very early stage of tumor. Patients with smaller tumors have a higher chance to be cured. Recent innovations in cancer prevention and detection have come to the molecular level to allow for a more accurate identification of at-risk individuals (4). Introduction of new drugs and treatment regimens, such as adjuvant chemotherapy, hormonal therapy and immunotherapy, prolongs the survival of BC patients, especially for the patients at advanced cancer stage. BC screening is not yet a common routine practice in each province in China. Most BC patients have already reached the middle or late stage at the time of diagnosis, which may explain why the mortality of BC is still increasing and why 5-year-survival rate of BC in China is lower than that in the United States (82% VS 90%) (5).

Common screening modalities for BC include palpation, blood-based assay and medical imaging methods (6). Palpation can be further divided into self-breast examinations (SBE) and clinical breast examinations (CBE) (6). SBE was used to be considered as the first line of BC screening, but results of two clinical trials failed to show a BC mortality benefit due to SBE (7, 8). On the contrary, SBE usually leads to self-panic and unnecessary testing or biopsies, which turns out to be harmful for the subject (6). CBE is a palpation performed by trained clinic staff. CBE has a high specificity of 0.94-0.99, but a very low sensitivity of 0.21-0.54 (6). Therefore, CBE cannot exclude the presence of BC. Blood-based assay is a non-invasive method to detect serum BC specific biomarkers. Suggested biomarkers, such as carcinoembryonic antigen (CEA) and cancer antigen 15-3 or 27-29 (CA15-3, CA27-29), usually lack sensitivity and/or specificity, and thus not suitable for early disease detection (9).

Diagnostic imaging modalities recommended by American Joint Committee on Cancer (AJCC) for BC screening include mammography (MMG), ultrasound (US) and magnetic resonance imaging (MRI). MMG uses low dose X-rays to examine lesions in the breast, allowing the examinations of small calcification points, tumor *in situ* (Tis), and structure of the breast. MMG sensitivity for BC declines significantly with increasing breast density (10). Breast US is a non-invasive examination that uses high-frequency acoustic reflection to reveal tissue inside the breast. Breast US is often used in conjunction with MMG to increase the sensitivity of BC detection for women at average risk. US can help to identify whether the lumps found in MMG is solid or filled with fluid. Although US is more accurate than MMG in differentiating breast masses or cysts, it cannot detect the small calcification points, the sign of early stage lesion, and is less sensitive to tumors with size less than 5 mm or deep in the breast. US also generates more false-positive examinations. MRI is often used in conjunction with MMG for high-risk women BC screening. MRI has no radiation exposure and can provide excellent images with high contrast and resolution under appropriate conditions. MRI is the most sensitive diagnostic tool for breast diseases because it can be performed in any direction without the influence of tissue overlap or breast composition. In the cost-effective aspect, MRI is more used in the diagnosing and staging process rather than BC screening. Aforementioned imaging methods can all detect early stage BC, however, these conventional imaging methods still have some limitations that would affect image quality and thus the diagnostic accuracy of the breast examination. Therefore, it is still in an urgent need to find a reliable biomarker allowing better screening and early diagnosis of BC.

Circulating tumor cells (CTC) are tumor cells shed from the primary tumor or metastatic sites into circulation. The 7th edition of AJCC Staging Manual for BC has introduced a new cancer stage, cM0(i+), at which no clinical or radiographic evidence of distant metastases is found, but tumors cells are still detected in the bone marrow, blood or distant non-regional lymph nodes. Therefore, CTC represent the process of tumor metastasis. CTC has been proven to be a prognostic factor in BC to predict patients’ survival outcomes (11, 12). Patients with metastatic BC (MBC) usually have more CTC and BC patients with more CTC usually have shorter progression free survival and overall survival (11). In addition, studies show as well that CTC can reflect the tumor burden and can be used as a monitoring biomarker to assess patients’ response to treatment and tumor recurrences (13). CTC are rare cells in the bloodstream. The most common strategy to enrich and identify CTC from the surrounding blood cells is based on the epithelial cell biomarkers. CellSearch^®^ (Menarini Silicon Biosystems, Huntingdon Valley, US), the only U.S. Food and Drug Administration (FDA) approved CTC system, use epithelial cell adhesion molecule (EpCAM) and cytokeratin (CK) antibody to enrich and identify CTC. Blood cells would not express EpCAM or CK, while most of the epithelial cells found in the circulation are tumor cells detaching from the solid tumors of epithelial origin. Therefore, it is common to use epithelial markers to detect CTC.

Previous studies showed CTC could reflect tumor burden in BC and can distinguish diseased patients from the healthy control (14). Overall CTC detection rate with CytoSorter^®^ (Hangzhou Watson Biotech, Hangzhou, China), a microfluidic-based immuno-capture CTC platform, in BC is 85.16%, and detection rates in early stage (stage I-II) BC are still more than 80%, suggesting that CTC could be used a diagnostic tool for BC screening (14). US and MMG are the two most common methods for BC screening in Chinese clinic. 238 BC patients, 217 patients with benign breast diseases (BBD), and 20 healthy females from 2 hospitals were enrolled in this study. We aimed to compare the performances of CTC, US and MMG in BC diagnosis and to assess whether the combination with CTC would enhance the diagnostic potency of US and MMG.

## Material and Methods

### Patients

In total, 238 female BC patients, including 17 ductal carcinoma *in situ* (DCIS), 82 stage I, 106 stage II, 31 stage III and 2 stage IV, 217 patients with BBD and 20 healthy females from Zhejiang University Medical College Affiliated Sir Run Shaw Hospital and Sun Yat-Sen University Sun Yat-Sen Memorial Hospital were enrolled in this study between December 2017 and December 2018. Control referred to patients with BBDs and healthy volunteers. Stage I-II BC patients were considered as patients at early stage. Inclusion criteria were as follows: (1) female patients age between 18 to 75 years; (2) patients had negative history of malignancy and were treatment-naive before enrollment; (3) patients received US and MMG examinations before diagnosis. (4) healthy females had no medical history of any malignant disease and no findings in breast by palpation, US and/or MMG. Exclusion criteria were as follows: (1) patients were pregnant or breast-feeding; (2) patients were currently undergoing or had prior cancer treatment; (3) patients had other malignant tumors or other malignant diseases within 5 years prior to enrollment; (4) patients had other conditions which investigators thought not suitable for the study. Patients’ clinicopathological characteristics, including age, menstrual state, histological type, grade, hormone receptors, human epidermal growth factor receptor 2 (HER2) and the clinical stage at diagnosis were collected.

### Blood Collection and CTC Detection

CTC were enriched by CytoSorter^®^ epithelial cells detection kit. CTC detection procedure was following CytoSorter^®^ manufacturer protocol and was described in the previous study (14, 15). In brief, the CytoChipNano was first coated with EpCAM antibody before placing onto CytoSorter^®^. The first 2 mL of peripheral blood was discarded to avoid potential skin epithelial cell contamination from venipuncture and 4 mL of blood was proceed to gradient-centrifuge within 6 hours after collection to collect the peripheral blood mononuclear cells (PBMC) layer. PBMC sample solution was then transferred into the spiral sample tube. Once the CTC enrichment was finished, the CytoChipNano was removed from CytoSorter^®^ and proceed to the immunofluorescence staining of PanCK-FITC (pan-cytokeratin-fluorescein isothiocyanate), CD45-PE (cluster of differentiation 45-phycoerythrin) and DAPI (4,6-diamidino-2-phenylindole). An OPPNO immunofluorescence microscopy (DSY5000X, OPPNO, Chongqing, China) was used to identify CTC by searching for PanCK-FITC+, CD45-PE-, and DAPI+ cells.

### Medical Imaging Examinations

US examination was performed with IU Elite^®^ (Philips Healthcare, Best, Netherlands). MMG examination was performed using Selenia^®^ (Hologic, Santiago, USA). Examination results were evaluated by experienced radiologists according to American College of Radiology (ACR) Breast Imaging-Reporting and Data System (BI-RADS) assessment categories.

### Statistical Analysis

Statistical analyses were performed using Prism 5.0 (Graphpad, La Jolla, CA, USA) and SPSS 20 (IBM, Armonk, NY, USA). Student t test was used for continuous variables, as appropriate. The *x^2^* test and Fisher’s exact test were used for the comparison of categorical parameters. One-way analysis of variance (ANOVA) was performed to calculate the differences among multiple groups. The receiver operating characteristic (ROC) curves were plotted to evaluate the sensitivity, specificity and area under the curve (AUC) value of the diagnostic methods. CTC, US and MMG cut-off values were determined by the highest Youden index (sensitivity + specificity - 1). A two-sided p value less than 0.05 was considered statistically significant.

### Combinational ROC Model

Combinational ROC analyses were performed using SPSS 20. The multiple variable were combined using logistic regression. In brief, the BI-RADS categories, 1, 2, 3, 4a, 4b, 4c, 5 and 6, were first convert into scores, 1, 2, 3, 4, 5, 6, 7 and 8, respectively. Binary logistic regression analysis was used to calculate the correlation coefficient of each variable in the combination with respect to diagnosis. Combination score would be obtained based on the variables and correlation coefficients. ROC of combination scores was drawn. The point closest to the left upper corner of the combinational ROC would be used as the combination score cut-off to calculate the sensitivity and specificity.

## Results

### CTC Can Reflect BC Patients’ Tumor Burden and Can Be Used as a Diagnostic Aid for BC Screening

**Figure 1:**
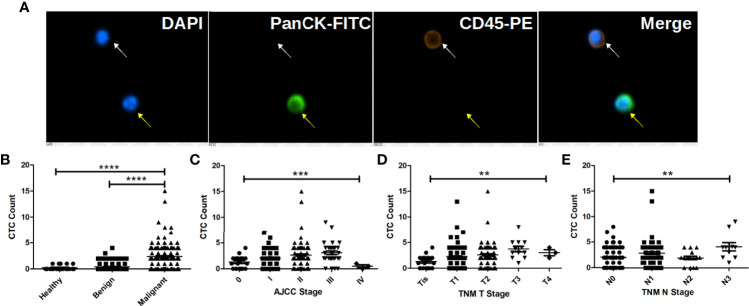
CTC are correlated with BC patients’ cancer stage, tumor size and lymph node involvement and can be used to distinguish BC patients from patients with benign tumors and healthy female. **(A)** Immunofluorescent staining of a captured CTC, indicated by the yellow arrow. CTC is defined as a DAPI (blue) positive, PanCK-FITC (green) positive and CD45-PE (orange) negative cell, while a white blood cell is indicated by the white arrow as a DAPI positive, CD45-PE positive and PanCK-FITC negative cell. **(B)** CTC enumeration can differentiate BC patients from patients with benign tumors and healthy females (both p < 0.0001). **(C)** CTC enumerations are correlated with BC patients’ cancer stage (p = 0.0007), tumor size (p = 0.0015) and lymph node involvement (p = 0.0034). More CTC are found in patient with bigger tumors and more lymph node involvement as shown in **(D, E)**. **** indicates P < 0.0001, *** indicates 0.0001< P < 0.001, while ** indicates 0.001 < P < 0.01.

CTC were identified as PanCK positive, CD45 negative and DAPI positive cells as shown in **Figure 1A**. Correlation of CTC with patients’ clinicopathological features are listed in **Table 1**. CTC are detected in 199 out of 238 BC patients, 71 out of 217 patients with BBD and 5 out of 20 healthy females. The average CTC counts (maximum CTC count) are 2.38 (15), 0.43 (4) and 0.25 (1), respectively. Most control (patients with BBD and healthy females) have less than 2 CTC. Statistical results show that CTC enumeration could be able to differentiate BC patients from patients with BBD and healthy females as shown in **Figure 1B** (both p < 0.0001). Statistical results show as well that CTC are correlated with AJCC stage (p = 0.0007), tumor size (p = 0.0015) and lymph node involvement (p = 0.0034) as shown in **Figures 1C–E**. Patients at advanced cancer stage or with bigger tumors or more lymph node involvement tend to have more CTC. CTC detection rates in early stages (stage I & II) BC patients are 82.93% and 86.79%, respectively. Furthermore, CTC are detected in 11 out of 17 Tis (DCIS) patients. Overall CTC detection in BC patients is 83.61%. Taken together, the results suggest that CTC could be used as a diagnostic tool for BC screening.

**Table 1:** Correlations of CTC with patients’ pathoclinical characteristics.

Group	n	Average Age (Median, Range) (years)	CTC detected in	CTC Detection Rate (%)	Average CTC Count (Range) (/4 mL)	p Value^*^
BC Patients	238	52.34 (51, 29-75)	199	83.61	2.38 (0-15)	**<0.0001**
Patients with BBD	217	46.97 (45, 20-73)	71	32.72	0.43 (0-4)	
Healthy volunteers	20	50.58 (49, 29-67)	5	25	0.25 (0-1)	
(All BC patients number = 238)						
***AJCC Stage***						**0.0007**
0	17	51.75 (46, 40-67)	11	64.71	1.29 (0-4)	
I	82	54.6 (55, 31-73)	68	82.93	2.06 (0-7)	
II	106	51.91 (51, 29-73)	92	86.79	2.63 (0-15)	
III	31	48.37 (46, 29-75)	27	87.1	3.06 (0-9)	
IV	2	48.5 (48.5, 34-63)	1	50	0.5 (0-1)	
***TNM Stage***						
***Tumor Size***						**0.0015**
Tis	17	51.75 (46, 40-67)	11	64.71	1.29 (0-4)	
T1	117	53.49 (54, 31-73)	98	83.76	2.23 (0-13)	
T2	91	51.52 (50, 29-75)	77	84.62	2.59 (0-15)	
T3	11	50.28 (54, 29-64)	11	100	3.73 (1-8)	
T4	2	38 (38, 37-39)	2	100	3 (2-4)	
***Lymph Node Involvement***						**0.0034**
N0	142	53.76 (53, 29-73)	111	78.17	2.06 (0-8)	
N1	72	50.99 (50.5, 29-69)	66	91.67	2.86 (0-15)	
N2	15	46.49 (45, 33-75)	11	73.33	1.93 (0-4)	
N3	9	50.52 (54, 34-64)	9	100	4.11 (1-9)	

CTC, circulating tumor cell; n, number of patients; BC, breast cancer; BBD, benign breast diseases; AJCC, American Joint Committee on Cancer; TNM, tumor-node-metastasis; Tis, tumor in situ.

^*^The p value of comparisons is based on the CTC enumeration of each group.

Bold values mean statistical significances.

### Comparison of Diagnostic Potency of CTC, US and MMG in BC Diagnosis

**Figure 2:**
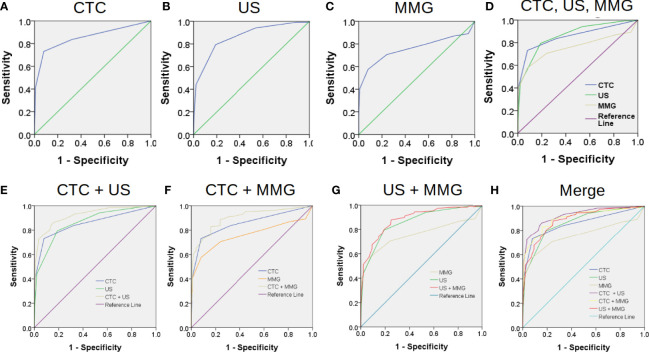
Combination of conventional medical imaging examinations with CTC enhances the diagnostic efficiency for BC. The performances of CTC, US and MMG in BC diagnosis are shown in **(A–C)** by the receiver operating characteristic (ROC) curves. Area under the curve (AUC) of CTC, US and MMG are 0.855, 0.861 and 0.759, respectively. CTC exhibits a similar diagnostic performance as US as shown in **(D)**. Combination of CTC enhances the performances of US and MMG in BC diagnosis as shown in **(E, F)** with AUC increasing from 0.855 to 0.922 and 0.759 to 0.899, respectively. However, combination of MMG with US does not improve the diagnostic performance of US much as shown in **(F)** with AUC increasing slightly from 0.855 to 0.884. “CTC + US” gives the best diagnostic performance, while “CTC + MMG” and “US + MMG” have similar improved diagnostic performances as shown in **(H)**.

The most common methods for BC screening in clinic are MMG and US. Therefore, we compared the performances of CTC, US and MMG in BC diagnosis. First, ROC curves were plotted separately as shown in **Figure 2**. When CTC cut-off value was set to 2, and both US BI-RADS and MMG BI-RADS cut-off scores were set to 4b, the highest Youden index of 0.65, 0.65 and 0.5 would be generated for CTC, US, and MMG, respectively. Detailed diagnostic performances of CTC, US and MMG are listed in **Table 2**. Among these three methods, US shows the highest sensitivity of 0.79. CTC and MMG have the same specificity of 0.92, while CTC shows the highest accuracy of 0.83. Based on the AUC, CTC exhibits a similar diagnostic potency as US.

**Table 2:** Diagnostic power of CTC, US and MMG in breast cancer diagnosis*.

	CTC	95% CI	US	95% CI	MMG	95% CI
Sensitivity	0.73	0.67 - 0.79	0.79	0.74 - 0.84	0.58	0.51 - 0.64
Specificity	0.92	0.88 - 0.95	0.81	0.75 - 0.86	0.92	0.88 - 0.95
Accuracy	0.83	0.79 - 0.86	0.80	0.76 - 0.84	0.75	0.71 - 0.79
Positive Likelihood Ratio (LR+)	9.12	5.88 - 14.13	4.18	3.19 - 5.48	7.18	4.60 - 11.20
Negative Likelihood Ratio (LR-)	0.29	0.24 -0.36	0.25	0.20 - 0.33	0.46	0.40 - 0.54
Youden Index	0.65	N/A	0.65	N/A	0.50	N/A
Area Under Curve (AUC)^#^	0.855	0.819 - 0.890	0.861	0.828 - 0.894	0.759	0.712 - 0.805
	**CTC + US**	**95% CI**	**CTC + MMG**	**95% CI**	**US + MMG**	**95% CI**
Sensitivity	0.90	0.86 - 0.94	0.87	0.82 - 0.91	0.87	0.82 - 0.91
Specificity	0.76	0.70 - 0.81	0.85	0.80 - 0.89	0.79	0.73 - 0.84
Accuracy	0.83	0.79 - 0.86	0.86	0.83 - 0.89	0.83	0.79 - 0.86
Positive Likelihood Ratio (LR+)	3.69	2.94 - 4.63	5.89	4.32 - 8.03	4.12	3.21 - 5.30
Negative Likelihood Ratio (LR-)	0.13	0.09 - 0.19	0.15	0.11 - 0.21	0.17	0.12 - 0.23
Youden Index	0.66	N/A	0.72	N/A	0.66	N/A
Area Under Curve (AUC)^#^	0.922	0.898 - 0.946	0.899	0.870 - 0.928	0.884	0.855 - 0.914

CTC, circulating tumor cell; CI, confidence interval; US, ultrasound; MMG, mammogram; N/A,. not applicable.

Confidence intervals for sensitivity, specificity and accuracy are “exact” Clopper-Pearson confidence intervals. Confidence intervals for the likelihood ratios are calculated using the “Log method”.

^*^CTC, US and MMG cut-off values were determined by the highest Youden index (sensitivity + specificity - 1). When CTC cut-off value was set to 2, and both US BI-RADS and MMG BI-RADS cut-off scores were set to 4b, the highest Youden index of 0.65, 0.65 and 0.5 would be generated for CTC, US, and MMG, respectively. Subjects with more than 2 CTC, or with US or MMG BI-RADS score higher than 4b are classified as CTC, US and MMG positive for diagnosis, respectively. As long as any measurement of composing parameter was higher than its cut-off value, the combination result would be considered as positive for diagnosis.

^#^AUC was determined by the ROC.

**Figure 3:**
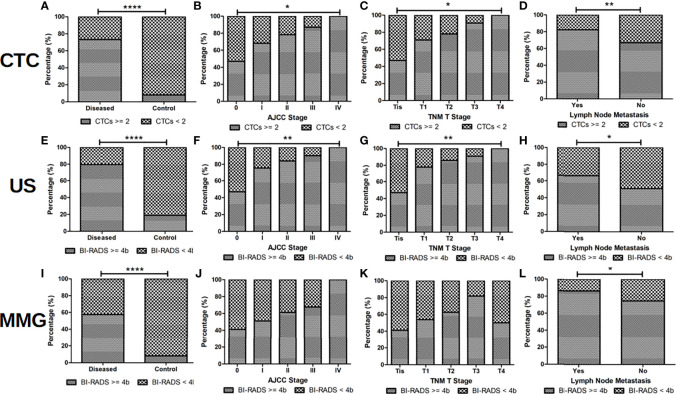
CTC and US are more sensitive than MMG for BC diagnosis. When CTC cut-off value of 2, US and MMG BI-RADS cut-off score of 4b were set, positive rates of CTC, US and MMG show significant differences between BC patients and controls (patients with BBDs and healthy volunteers) as shown in **(A, E, I)**, respectively (all p < 0.0001). US should be slightly more sensitive than CTC in BC diagnosis, for positive rates of US are more correlated with BC patients’ cancer stage and tumor size than CTC as shown in **(B, C, F, G)**. No statistical significance is found between positive rate of MMG and BC patients’ cancer stage and tumor size as shown in **(J)** and **(K)**. Positive rates of CTC, US and MMG showed significant differences between BC patients with and without lymph node metastases as shown in **(D)**, **(H)** and **(L)** (p = 0.0086, 0.0271 and 0.0195, respectively). **** indicates P < 0.0001, ** indicates 0.001< P < 0.01, while * indicates 0.01 < P < 0.05.

Subjects with more than 2 CTC, or with US or MMG BI-RADS score higher than 4b are classified as CTC, US and MMG positive for diagnosis, respectively. Correlation of CTC, US and MMG positive rates with patients’ clinicopathological characteristics are listed in **Table 3** and shown in **Figure 3**. CTC, US and MMG positive rates show significant differences among BC patients, patients with BBD and healthy females (all p < 0.0001). CTC and US positive rates are associated with cancer stage (p = 0.0021 and 0.0034, respectively), tumor size (p = 0.0444 and 0.0054, respectively) and lymph node metastases (p = 0.0086 and 0.0271, respectively), while MMG positive rate is only associated with lymph node metastases (p = 0.0195). For early stage BC diagnosis, US has the highest sensitivity of 0.80, followed by CTC (0.74) and then MMG (0.57). Taken together, CTC and US have similar performances in BC diagnosis, and MMG shows the least diagnostic potency.

**Table 3:** Clinicopathological characteristics of BC patients with ≥ 2 or < 2 CTC, with US BI-RADS score ≥ 4b or < 4b, and with MMG BI-RADS score ≥ 4b or < 4b.

Groups	n	CTC		p Value^*^	US BI-RADS		p Value^*^	MMG BI-RADS		p Value^*^
		≥ 2	< 2		≥ 4b	< 4b		≥ 4b	< 4b	
BC Patients	238	174	64	**<0.0001**	189	49	**<0.0001**	137	101	**<0.0001**
Patients with BBD	217	19	198		45	172		19	198	
Healthy volunteers	20	0	20		0	20		0	20	
(All BC patients number = 238)										
***AJCC Stage***				**0.0021**			**0.0034**			0.1573
***0***	17	8	9		8	9		7	10	
I	82	56	26		62	20		42	40	
II	106	83	23		89	17		65	41	
III	31	27	4		28	3		21	10	
IV	2	0	2		2	0		2	0	
***Tumor Size***				**0.0444**			**0.0054**			0.1857
Tis	17	8	9		8	9		7	10	
T1	117	83	34		91	26		63	54	
T2	91	71	20		78	13		57	34	
T3	11	10	1		10	1		9	2	
T4	2	2	0		2	0		1	1	
***Lymph Node Metastasis***				**0.0086**			**0.0271**			**0.0195**
Yes	96	79	17		83	13		64	32	
No	142	95	47		106	36		73	69	

BC, breast cancer; CTC, circulating tumor cell; US, ultrasound; BI-RADS, breast imaging-reporting and data system; MMG, mammogram; n, number of patients; BBD, benign breast diseases; AJCC, American Joint Committee on Cancer; Tis, tumor in situ.

^*^Based on the Youden index analysis, CTC, US and MMG cut-off values were 2, 4b, and 4b, respectively. Subjects with more than 2 CTC, or with US or MMG BI-RADS score higher than 4b are classified as CTC, US and MMG positive for diagnosis, respectively. The p value of comparisons is based on the positive proportion among groups.

Bold values mean statistical significances.

### Combination With CTC Improves the Performances of US and MMG in BC Diagnosis

Next, we investigated whether the combination with CTC would enhance the performances of US and MMG in BC diagnosis. As shown in **Table 2** and **Figure 2**, combining with CTC increases the AUC of US and MMG from 0.861 to 0.922 and 0.759 to 0.899, respectively, while combining with MMG increases only slightly the AUC of US from 0.861 to 0.884. Although US has a slightly higher AUC of 0.861 than CTC (0.855), the combination of CTC with MMG generates a higher AUC of 0.899 than the conjugation of US with MMG (0.884). As long as any measurement of composing parameter was equal to or higher than its cut-off value, the combination result would be considered as positive for diagnosis. Combination with CTC increases the diagnostic sensitivity of US and MMG from 0.79 to 0.90 and 0.58 to 0.87, respectively. But at the same time, the specificity decreases from 0.81 to 0.76 and 0.92 to 0.85, respectively. Among these three combinations, “CTC + US” has the highest AUC of 0.922, followed by “CTC + MMG” (0.899) and then “US +MMG” (0.884). However, “CTC + MMG” has the highest accuracy of 0.86, followed by “CTC +US” (0.83) and “US + MMG” (0.83). Combination with CTC or US increases the sensitivity of MMG both to 0.87, but “CTC + MMG” has a higher specificity of 0.85.

As shown in **Table 4**, due to decreased specificity caused by the combinations, “CTC + US”, “CTC + MMG” and “US +MMG” all show less statistical correlation with the patients’ clinicopathological features. As for early stage BC diagnosis, “CTC + US”, “CTC + MMG” and”US +MMG” have sensitivities of 0.91, 0.87 and 0.88, respectively. Taken together, combining with CTC would improve the performances of US and MMG in BC diagnosis, especially for MMG. Regarding improved diagnostic performance, the combination of CTC with either of US or MMG is better than the combination of US and MMG together.

**Table 4:** Clinicopathological characteristics of BC patients diagnosed by CTC combined with US, CTC combined with MMG, or US combined with MMG.

Groups	n	CTC + US		p Value^#^	CTC + MMG		p Value^#^	US + MMG		p Value^#^
		Positive^*^	Negative		Positive^*^	Negative		Positive^*^	Negative	
BC Patients	238	215	23	**<0.0001**	207	31	**<0.0001**	207	31	**<0.0001**
Patients with BBD	217	58	159		35	182		50	167	
Healthy volunteers	20	0	20		0	20		0	20	
(All BC patients number = 238)										
***AJCC Stage***				**0.0137**			0.0951			**0.03**
0	17	12	5		12	5		11	6	
I	82	71	11		68	14		69	13	
II	106	100	6		96	10		96	10	
III	31	30	1		29	2		29	2	
IV	2	2	0		2	0		2	0	
***TNM Stage***										
***Tumor Size***				0.0662			0.119			0.0695
Tis	17	12	5		12	5		11	6	
T1	117	106	11		99	18		102	15	
T2	91	85	6		84	7		82	9	
T3	11	10	1		10	1		10	1	
T4	2	2	0		2	0		2	0	
***Lymph Node Involvement***				**0.0115**			0.0596			0.1872
N0	142	121	21		118	24		118	24	
N1	72	71	1		68	4		67	5	
N2	15	14	1		12	3		14	1	
N3	9	9	0		9	0		8	1	
***Lymph Node Metastasis***				**0.0011**			**0.0307**			**0.0307**
Yes	96	94	2		89	7		89	7	
No	142	121	21		118	24		118	24	

BC, breast cancer; CTC, circulating tumor cell; US, ultrasound; MMG, mammogram; n, number of patients; BBD, benign breast diseases; AJCC, American Joint Committee on Cancer; TNM, tumor-node-metastasis; Tis, tumor in situ.

^*^Based on the Youden index analysis, CTC, US and MMG cut-off values were 2, 4b, and 4b, respectively. As long as any measurement of composing parameter was higher than its cut-off value, the combination result would be considered as positive for diagnosis.

^#^The p value of comparisons is based on the positive proportion among groups.

Bold values mean statistical significances.

## Discussions

Although our results confirmed the previous findings that CTC could be used to distinguish BC patients from the healthy females or patients with BBD (both p < 0.0001) and CTC could reflect tumor burden (14), still 71 out of 217 patients with BBD had CTC detected. We used epithelial markers to detect CTC and the identified cells were not further validated by any tumor specific marker. Therefore, the CTC found in patients with BBD were not truly tumor cells but circulating epithelial cells. A study with CellSearch^®^ system showed as well that positive events that met the criteria for “CTC” were detected in 11.3% of patients with benign colon diseases (16). Our results showed that CTC are correlated with tumor stage, tumor size and lymph node involvement (p= 0.0007, 0.0015 and 0.0034, respectively. Clinical value of CTC in BC has started to be recognized gradually by most BC experts worldwide. In the 8th edition of the AJCC BC guidelines published in 2018, it is written that CTC can be used as a prognostic biomarker to predict patients’ survival outcomes. In the 2019 Chinese Society of Clinical Oncology (CSCO) BC clinical guidelines, it is written that CTC can reflect the condition of tumor tissue and can be used as a replacement of biopsy samples for pathological diagnosis, disease monitoring and molecular sequencing. CTC can be used to monitor treatment response and to predict prognosis (11–13). CTC has been proposed as a screening tool for lung cancer in high-risk groups of people (17). Based on the reviews of CTC in BC published in 2013 and 2016, maximum CTC detection rates in early stage and metastatic BC are 55% and 54%, respectively (11, 18). The detection rate varied depending on which CTC enrichment method was used (11, 18). CellSearch^®^ system has CTC detection rates in BC less than 40% (18). Metastasis is an inevitable process during tumor progression. According to the recent study on ex vivo colorectal tumor model, a CTC can be released into circulation even when the tumor is smaller than 0.01 cm^3^ (19). CTC represents the process of metastasis. Just due to the heterogeneous tumor nature, some patients would have less CTC in the circulation. In theory, as long as a detection method is sensitive, CTC should be detected in every cancer patient. Thus, CTC should be a reliable method for cancer screening. The reason why CTC has not been suggested as a screening tool for BC in practice might be due to the low detection rates. However, with the improvements of the CTC enrichment techniques recently, CTC detection is getting more sensitive. Liang et al. used CanPatrol™ system to detect CTC in early stage BC, and the detection rate is 81% (20). We used CytoSorter^®^ to detect CTC in BC, and the overall CTC detection rate is 83.61% and the detection rate in early stage is 84.86%. With such a high detection rate, CTC can no doubt be used as a screening tool for BC. But a breast cancer specific marker might be required to be ascertained that the captured CTC originate from breast lesions and to reduce the false positive results in patients with BBD and healthy people.

Our results suggest that CTC can reflect tumor burden. BC patients at advanced stage, with bigger tumor and more lymph node involvement have usually more CTC. But in fact, less CTC were detected and a lower CTC detection rate was found in stage IV patients. It could be due to that the sample size is too small (n = 2) or we used the wrong antibody to capture CTC. EpCAM antibody is supposed to capture the epithelial type of CTC. According to the AJCC BC staging guidelines, any TxNx patient with distant metastasis is classified as a stage IV patient. Epithelial-mesenchymal transition (EMT) plays an important role during tumor metastasis (21, 22). An epithelial type of CTC must transform into a migratory mesenchymal CTC before it settles down to a distant site. During EMT, cells lose expression of epithelial markers, such as EpCAM or CK, which might explain why less CTC were detected in stage IV BC patients with EpCAM antibody (22). Studies have shown patients with more mesenchymal type of CTC usually have worse survival outcomes (22, 23). However, further studies need to be conducted to confirm whether BC patients at advanced stage, with bigger tumors, or more lymph node involvement would have more mesenchymal type of CTC.

US and MMG are the two most common screening tools for BC in China. Next, we compared the diagnostic potency of CTC, US and MMG for diagnosing BC, especially in the detection of early stage BC. As shown in **Tables 2** and **3**, US has the highest sensitivity of 0.79, followed by CTC (0.73) and then MMG (0.58). CTC and MMG have the same specificity of 0.92, followed by US (0.81). The AUC of CTC, US and MMG are 0.855, 0.861 and 0.759, respectively. CTC has the highest accuracy of 0.83, followed by US (0.80) and then MMG (0.75). As for early stage tumor diagnosis, US has the highest sensitivity of 0.80, followed by CTC (0.74) and then MMG (0.57). Based on the AUC, CTC and US have similar diagnostic potency. Although the sensitivity of US is higher, it generates more false-positives as well, which might lead to over-diagnosis and panic in patients with BBD. The specificity and accuracy of CTC are slightly higher than those of US. Taken together, in our study, CTC performs the best in BC diagnosis, followed by US and then MMG. There are two limitations that restrain the use of CTC in routine practice as a diagnostic aid. First, a standard for CTC is still lacking. Many techniques have been developed to enrich CTC. Methodologies with low sensitivities are not suitable for clinical use. But for the ones with high sensitivities, results of different methods are sometimes not comparable with each other. Thus, a standard must be established before CTC can be used as a common diagnostic tool. Second, comparing to other diagnostic tools in clinic, CTC is pricey and usually not covered by the health insurances. High cost limits the clinical use of CTC in practice.

MMG is in fact the gold standard for BC screening, and is the only screening modality that has shown to lead to a reduction in BC mortality (24). Screening MMG leads to a 19% overall reduction in BC mortality (25). However, the sensitivity of MMG depends on the patients’ age and breast composition (26). MMG is more sensitive in women over 50 than in younger women, and in women with fatty breasts than ones with dense breasts (27). Regarding BC mortality, screening MMG is less beneficial for women in their 40s (15%) and more useful for women in their 60s (32%) (25). Sensitivity of MMG was 0.82 among women with predominantly fatty breast, but 0.24 in women with heterogeneous dense breasts (28). As older women tend to have fatty breasts, we analyzed the diagnostic potency of MMG in BC diagnosis among different BC patients grouped by age and found that sensitivity and AUC of MMG increased in older women (data not shown). Furthermore, Chinese women usually have dense breasts (29). Taken together, the dense breast should be the reason why MMG has the lowest diagnostic sensitivity in our study. The sensitivity of US depends on the patient’s age and breast composition as well (28). US usually has a higher sensitivity than MMG in women younger than 45 years, whereas MMG has a higher sensitivity than US in women older than 60 years (28). But in our study, US showed higher sensitivities of BC detection in both women younger than 45 years and older than 60 years (data not shown). Sensitivity of US was 0.71 among women with predominantly fatty breast and 0.57 for heterogeneous dense breasts (28). In a cohort study of 30-39 years old women, US showed a better sensitivity of 0.96 compared to MMG (0.61) (30). However, MMG had a better specificity of 0.94 compared to 0.89 for US (30). The use of US in conjugation with MMG increase both sensitivity and specificity for BC screening (28). In another single-center, prospective, non-randomized comparison study, Cortesi et al. found that MRI, MMG and US had different diagnostic sensitivity in different group of people (31). In BRCA mutated patients, MRI alone with annual US could be offered. In high risk patients, MMG plus biannual US provide the most sensitive diagnosis and for intermediate risk group an annual MMG could be sufficient (31). Overall, the most sensitive technique was MRI (0.94) followed by MMG (0.55) and US (0.29) (31). Berg et al. shows that US is comparable with MMG for BC screening, and US is more sensitive for invasive and node-negative cancers (32). Common limitation for US and MMG is the false positives. In our study, US has a higher false positive rate of 19% than MMG (8%), which is consistent with previous study showing that false positive are more common with US screening (32).

MMG is the gold standard for BC screening recommended by American Cancer Society, but MMG shows the lowest sensitivity of 0.58 in our study. Lastly, we investigated whether combination with CTC would increase the sensitivity of MMG and at the same time maintain the specificity in an acceptable level. As shown in **Table 2**, the combination with CTC enhances the diagnostic performances of US and MMG indicated by the increased AUC. Among these three combinations, “CTC + US” has the highest AUC and sensitivity of 0.922 and 0.90, respectively. “CTC + MMG” has the highest specificity and accuracy of 0.85 and 0.86, respectively. The combination with CTC or US increases the sensitivity of MMG by 50% to 0.87, but “CTC + MMG” has a higher specificity of 0.85 than “US + MMG” of 0.79. Therefore, “CTC + MMG” performs better than “US + MMG” in BC diagnosis. Based on the AUC, combination improves the diagnostic performance. “CTC + US” has the best performance, followed by “CTC + MMG” and then “US + CTC”. Based on the specificity and accuracy, “CTC + MMG” is the best combination. Theoretically, we should use the point closest to the left upper corner of the combinational ROC as the combination score cut-off to calculate the sensitivity and specificity. However, this model would be too complicated to be used in practice. In practice, the combinational results would be considered as positive as long as either one of composing measurements is higher than its cut-off or all of the composing measurements are higher than cut-off. We used the former definition in our study. When CTC is more than 2 or US/MG BI-RADS is higher than 4b, the combination result would be considered as positive. The sensitivity would usually increase while specificity would decrease in this model. However, it still fit the combinational ROC as shown in **Figures 2E–G**. The points closest to the upper left corners of the curves had higher sensitivities and slightly lower specificity. Since previous study (32) and our results showed US generated falser positive, it would be more logic to define the positive result for “CTC + US” as CTC ≧ 2 and BI-RADS ≧ 4b at the same time. In this definition, “CTC + US” has a sensitivity, specificity and accuracy of 0.62, 0.97 and 0.80, respectively. The specificity of US is much improved in this model. Thus, in practice, we can choose which model to be used depending on sensitivity or specificity we want to improve. MMG usually has a low sensitivity for BC diagnosis, therefore, it would be better to use the definition of positive result for “CTC + MMG” as CTC ≧ 2 or BI-RADS ≧ 4b. Our results indicate “CTC + US” or “CTC + MMG” performs better than “US + MMG” in BC diagnosis.

Our study is the first study comparing the diagnostic performances of CTC, US and MMG in the same cohort. Results of this work show that CTC detected by CytoSorter^®^ can be used as a diagnostic aid to assist in early diagnosis and screening of BC. Combination of CTC enhances the diagnostic efficiency of US and MMG for BC screening, especially for MMG in Chinese women. Still more studies on larger patient population should be conducted to confirm our findings.

## Data Availability Statement

The raw data supporting the conclusions of this article will be provided by the authors on request.

## Ethics Statement

The studies involving human participants were reviewed and approved by the ethics committees of Zhejiang University Medical College Affiliated, Sir Run Run Shaw Hospital, and Sun Yat-Sen University, Sun Yat-Sen Memorial Hospital, with IRB number, Qi Xie Lin Chuang Shi Yan 20180427-1 and [2018] kuaishendi(75)Hao, respectively. The patients/participants provided their written informed consent to participate in this study.

## Author Contributions

Conception and design: YG and XK. Acquisition of data: WZ, JZ, and CD. Analysis and interpretation of data: YG and W-HF. Writing of the manuscript: YG and W-HF. Review of the manuscript: All authors. All authors contributed to the article and approved the submitted version.

## Conflict of Interest

The authors declare that the research was conducted in the absence of any commercial or financial relationships that could be construed as a potential conflict of interest.
